# Differences in Method-Specific Vancomycin MICs and Induced Daptomycin Resistance in an Infective Endocarditis Patient

**DOI:** 10.1155/2015/175810

**Published:** 2015-05-03

**Authors:** David Benjamin Lash, Jeremiah Joson, Arash Heidari

**Affiliations:** ^1^Department of Pharmacy, Kern Medical Center, Bakersfield, CA 93306, USA; ^2^Department of Medicine, Kern Medical Center, Bakersfield, CA 93306, USA

## Abstract

Methicillin-resistant *Staphylococcus aureus* (MRSA) is a common nosocomial infection that has a high burden of morbidity and mortality. Vancomycin is the often-used antibiotic of choice when MRSA is suspected as a causative infectious agent. Recent studies have called into question the reliability of vancomycin as empiric therapy, especially in instances of bacteremia. The isolate's minimum inhibitory concentration (MIC), the source of infection, modality of susceptibility testing, and antibiotic resistance are all issues that should be taken into consideration when formulating a care plan for a patient. We present a case that illustrates some of these issues clinicians are facing.

## 1. Introduction

Health care-associated methicillin-resistant* Staphylococcus aureus* (MRSA) infections are a serious concern for health care providers as they are associated with high mortality rates [1]. The minimum inhibitory concentration (MIC) for MRSA isolates has received much attention as a potential risk factor for treatment failure and mortality. Blood stream infections (BSIs) specifically may be associated with poorer outcomes when MRSA isolates have a high (≥2 mg/L) MIC [2]. Having an elevated MIC results in a smaller area under the curve to MIC ratio (AUC/MIC), and this diminished exposure to vancomycin is a potential explanation of treatment failure [3]. Daptomycin is a therapeutic alternative to vancomycin in BSIs and some studies suggest that daptomycin may be superior to vancomycin, although randomized controlled trials are lacking [4]. The exact role of both of these antimicrobial agents continues to be refined as more evidence becomes available in the literature and the following case depicts many of the difficulties and uncertainties providers are facing.

## 2. Case

A 58-year-old male presented to the emergency department complaining of a large 8 cm × 8 cm parasternal growth on his chest that he first noticed 3 days previously. He denied injectable drug use (IDU). Blood cultures were collected prior to starting empiric vancomycin (1 g IV Q12Hr) and piperacillin/tazobactam (3.375 g IV Q6Hr).

On day 1 of admission, the parasternal growth was characterized as a phlegmon based upon computed tomography (CT) scans with contrast to the neck and chest; incision and drainage (I&D) was not performed. Computed tomography of the chest was notable for multiple pleural-based pulmonary nodules and degenerative changes in the cervical spine. Vancomycin was increased to 1.5 g IV Q12Hr. Repeat blood cultures were drawn on day 2.

On day 3, the patient admitted to recent injection drug use with his last use being 3 weeks ago. It was decided to treat him for suspected infective endocarditis (IE) due to bacteremia, pulmonary nodules suspicious of septic emboli, and IDU. Sensitivities of admitting blood cultures showed methicillin-resistant* Staphylococcus aureus* (MRSA) with a vancomycin MIC of 0.5 mg/L (VITEK 2, bioMérieux, Marcy l'Etoile, France), and a confirmatory Epsilometer test (Etest, AB BIODISK, Solna, Sweden) was ordered. A subtherapeutic vancomycin trough of 7.6 mg/L prompted a dose increase to 1.5 g Q8Hr. Piperacillin/tazobactam was discontinued. Transthoracic two-dimensional echocardiogram (TTE) showed a nondiagnostic density near the aortic valve; transesophageal echocardiography (TEE) was unremarkable. The patient also started complaining of lumbar spine tenderness. A lumbar X-ray showed no evidence of destructive osseous lesions. Vancomycin trough was 19.8 mg/L.

On day 6, I&D was performed on the patient's parasternal mass and cultures were taken. Etest MICs on the admitting blood culture came back as 0.5, 0.75, and 2 mg/L for ceftaroline, daptomycin, and vancomycin, respectively. Vancomycin was discontinued and daptomycin was initiated at a daily dose of 6 mg/kg (500 mg) intravenously. Blood cultures collected on day 5 were negative for growth.

On day 16, a right L5-S1 hemilaminotomy for biopsy was performed. The patient noted an immediate relief of back pain after the procedure. Repeat chest CT with contrast on day 18 showed a decrease in the number and size of pulmonary nodules. The patient was subsequently transferred to a skilled nursing facility (SNF) to complete 42 days of daptomycin treatment.

On day 20, cultures and sensitivities from the lumbosacral disc were reported with Etest MICs of 0.75, 4.0, and 2.0 mg/L for ceftaroline, daptomycin, and vancomycin, respectively. These results were relayed to the physician overseeing care of the patient at the SNF and the patient was switched to ceftaroline 600 mg intravenously twice a day to complete therapy.

## 3. Discussion

Reports of vancomycin treatment failure in isolates with an MIC of >1 mg/L, as well as toxicity concerns for MICs ≥2 mg/L, have caused experts to recommend alternative therapies in isolates with a vancomycin MIC ≥2 mg/L [5, 6]. While the reasoning behind avoidance of vancomycin in these patients has largely been due to pharmacokinetic concerns, several studies have given clinicians further cause for concern. A study by Holmes et al. found that high vancomycin MIC (defined as Etest >1.5 mg/L) was significantly associated with 30-day mortality regardless of whether the isolates were methicillin-sensitive or methicillin-resistant [7]. The authors found no correlation between oxacillin MIC and vancomycin MIC, further suggesting that vancomycin MIC may independently correlate to expected outcomes and may perhaps be a marker of the isolate's resilience to antibiotic therapy. Another study found that endocarditis and pneumonia, as sources of MRSA bacteremia, were associated with higher risk of treatment failure [8]. This study had a high (over 80%) percentage of isolates with MIC ≥1.5 mg/L, but the mounting body of evidence is enough to place serious concern as to whether vancomycin should be the empiric antibiotic of choice in IE. It should also be noted that the Infectious Diseases Society of America does give daptomycin a higher level of evidence rating in native valve IE as compared to vancomycin (A-I versus A-II) [6].

Daptomycin is strongly recommended as an agent in native valve IE, but it is not without its limitations as well, notably development of resistance. There are several genetic mutations that have been noted to be related to daptomycin resistance, but exact mechanisms of resistance have not been completely elucidated. One mechanism that has been suggested is that antecedent vancomycin exposure produces a thickened cell wall that serves as a physical barrier to daptomycin [9]. While antecedent vancomycin may induce resistance, there is also the possibility that the standard dose of 6 mg/kg is too low. High dose daptomycin (8–10 mg/kg) has been used effectively in both right sided infective endocarditis and left sided infective endocarditis, and there is some evidence to suggest that higher doses result in better outcomes in MRSA isolates with an MIC >1 mg/L [10, 11].

Clinicians should further be mindful of susceptibility testing in IE. There have been several studies conducted specifically on MRSA isolates comparing reported MICs by various methods, which found a tendency for the VITEK2 machine to underestimate MICs as compared to the Etest method [12, 13]. These findings are not insignificant as isolates with an Etest MIC of ≥2 mg/L are associated with treatment failure and a higher mortality rate compared to isolates with lower MICs [12, 14].

This case report illustrates the pitfalls of current empiric treatment regimens as well as relying on results reported via automated methods. Should vancomycin remain a mainstay of empiric therapy or should other antimicrobial options be utilized in our sicker patients? As the incidence of antimicrobial resistance increases, it is imperative that clinicians have the proper knowledge to face these challenges in patient care. The continued role of vancomycin as empiric therapy, the methods in which we conduct susceptibility testing, the safety and efficacy of high dose daptomycin, and the mechanisms of inducing antimicrobial resistance are all topics that warrant further discussion and research.
